# Longitudinal associations of perceived occupational challenges and depressive symptoms among middle-aged German workers

**DOI:** 10.1192/bji.2025.10086

**Published:** 2026-11

**Authors:** Paulina Sophie Witte, Hans-Helmut König, André Hajek

**Affiliations:** Department of Health Economics and Health Services Research, University Medical Center Hamburg-Eppendorf, Hamburg, Germany. Email: witte.paulina@icloud.com

**Keywords:** Overload, under-challenge, depressive symptoms, occupation, gender differences, mental health, depression, stress

## Abstract

**Background:**

Studies have demonstrated that high job strain and low job satisfaction can lead to depression. However, less focus has been recorded on the effects of a worker’s perceived challenges related to their qualifications.

**Aims:**

We aimed to investigate the association between perceived professional under-challenge or overload and depressive symptoms (also stratified by gender), based on nationally representative longitudinal data, thereby adding methodological novelty to previous cross-sectional research approaches.

**Method:**

This study used longitudinal data from the nationally representative German Ageing Survey covering community-dwelling individuals aged 40–64 years. The analytic sample included 7487 observations from 4362 individuals, spanning 4 survey waves (2008–2017). Key variables were depressive symptoms (measured with the 15-item Center for Epidemiologic Studies Depression Scale), perceived occupational challenge (via self-report) and relevant time-varying covariates (age, marital status, net household income, self-rated health, chronic diseases). Linear fixed-effects regressions were used to analyse longitudinal associations.

**Results:**

Fixed-effects regressions showed that transitions towards overload were significantly associated with increased depressive symptoms (*β* = 1.39, *P* < 0.01), while transitions towards not being sufficiently challenged showed no significant associations. When stratified by gender, similar patterns were observed for men, with significant associations between overload and increased depressive symptoms (*β* = 2.16, *P* = 0.004).

**Conclusion:**

Our study indicates that changes towards job overload are linked to increased depressive symptoms in middle-aged men, emphasising the importance of managing work challenges and fostering a healthy work environment for employees’ mental health.

Major depression represents a pervasive challenge within our society. The World Health Organization rated major depression as the third leading cause of the global burden of disease in 2008.^1^ In 2019, approximately 280 million individuals worldwide suffered from a depressive disorder, reflecting a prevalence of 3.8% in the global population. The prevalence of depression is higher in some age groups, with rates of 5.0% in adults aged 20 years and ranging from 5.2 to 5.8% in adults aged 40–69.^2^ Gender differences further compound this issue, because women are around twice as likely as men to develop a depressive episode, a divide that is apparent from young adulthood to the post-menopausal stage.^3^

The consequences of depressive disorders extend beyond individual suffering, impacting family, friends and the workplace. In severe cases, these can lead to suicidal thoughts and actions, with depression being a prominent factor in approximately 800 000 global suicides in 2015.^4^ In addition to the individual effects and reduced quality of life, depression also exacts an economic toll through reduced productivity and an increase in both sick leave days and direct costs.^5^

Considering the time that adults dedicate to work, it becomes imperative to identify work-related risk factors for the development and persistence of a depressive disorder. Because middle-aged individuals primarily drive our economy, prioritising their long-term health is critical. Simultaneously, given its pivotal role, we must address whether the workplace significantly impacts their mental health. In a meta-analysis published in 2005, it was elucidated that job dissatisfaction is significantly associated with mental disorders, particularly burnout and depression.^6^ Another recent meta-analysis has also shown a correlation between job strain and clinical depression.^7^ Despite considering the factors influencing job satisfaction – such as income, working hours, workload and interpersonal dynamics – the most recent literature largely fails to account for other relevant aspects, such as subjective under- or overload given the qualifications required for the work. Only a few studies have addressed subjective challenges.^8^ Recent meta-analytic evidence from a comprehensive global review of healthcare professionals highlights the significant and multifaceted relationships among job stress, low job satisfaction, workplace bullying and burnout, emphasising the importance of addressing both workload- and qualification-related challenges in occupational health research. While this meta-analysis almost exclusively includes cross-sectional and case-control studies that provide snapshots of associations between occupational stress and outcomes such as burnout, these designs cannot establish temporal sequences or causality and may be influenced by transient circumstances or confounding factors.^9^ The majority of studies examining job satisfaction, as well as the limited research on subjective overload and under-challenge in association with depressive symptoms, adopt a cross-sectional and observational design.^10,11^

For example, a cross-sectional study published in 2011 showed an association between subjective under-challenge and depressive symptoms.^8^ Another study, based on cross-sectional data, showed that mental health could be damaged by either too many or too few challenges compared with exactly the right level of challenges.^10^

To date, there is very restricted evidence regarding the association between self-rated under-challenge or overload at work and depressive symptoms. The studies mentioned above are restricted in their cross-sectional design, which makes it challenging to elucidate causality and directionality. However, they point to the importance of studying depressive symptoms more closely in connection with work-related factors. It is crucial to examine the complex relationship between the challenge levels in occupational settings and the manifestation of depressive symptoms. Given the absence of findings based on longitudinal data, this study aims to examine longitudinally, among middle-aged individuals, the association between subjective overload or under-challenge, given one’s qualification for the occupation, and the occurrence of depressive symptoms.

Understanding the potential link between inadequate subjective challenge at work and depressive symptoms may help to identify middle-aged individuals at risk for depression longitudinally, and thus the opportunity to intervene early. By employing a longitudinal analytic framework, this study allows for temporal inferences and captures intra-individual change, marking a methodological advancement over previous cross-sectional designs.

## Method

### Sample

This study utilised data gathered from waves 3 (2008), 4 (2011), 5 (2014) and 6 (2017) of the cross-sectional and longitudinal German Ageing Survey (DEAS). The survey was initiated in 1996 by the Research Data Centre of the German Centre of Gerontology (DZA), funded by the Federal Ministry for Family Affairs, Senior Citizens, Women and Youth. The survey sampled Germans registered within municipalities and who are in their ‘second half of life’, aged 40 years and above. Employing a cohort-sequential design, DEAS introduced new baseline samples approximately every 6 years (in waves 2 in 2002, 3 in 2008 and 5 in 2014); since 2008, the DEAS panel has been surveyed every 3 years.^12,13^

The DEAS study enrolled individuals through a national probability sampling to ensure representative inclusion. The adults included in the survey are a sample based on a random selection of 290 municipalities drawn in 1996 from the total of 12 000 municipalities that existed in Germany at that time. Each baseline year, the local population registries of these 290 municipalities served as the basis for sampling individuals aged between 40 and 85 years and who lived in private households within the community. The DEAS survey covers various topics such as socioeconomic circumstances, family dynamics, work and retirement, health-related aspects and age transition. The data were collected through standardised questionnaires administered by trained interviewers and self-administered written questionnaires. The participants in each wave were invited to complete future waves, ensuring longitudinal data.^12,13^ To reduce attrition, DEAS reduced the interval between panel waves from 6 to 3 years from 2008 onwards, and thus we have focused on the waves from 2008 until the beginning of the COVID-19 pandemic to ensure the highest possible follow-up. In 2008, a total of 17 366 individuals were eligible for inclusion and the response rate was 35.7%. The retention rate from the baseline year (2008) to the survey year (2011) was, for example, 46.1%, and the retention rate from 2008 to 2014 was 41.4%. Please see the DEAS Cohort Profile for further details.^12^

Due to the COVID-19 pandemic and the subsequent shifts in social and work behaviour – as well as the effects on mental health – we relied on data from waves 3–6, gathered before the onset of the pandemic and capturing all relevant variables. This study employed linear fixed-effects regressions to investigate intra-individual variable changes over time (please see Statistical analysis, below, for further details). Our analytical sample included 7487 observations from 4362 individuals. Stratified by gender, our analysis includes 3668 observations corresponding to 2169 individuals among males, and 3819 observations corresponding to 2193 individuals among females.

The DEAS study followed the Declaration of Helsinki but did not require an ethical declaration because the prerequisites were unmet. Because no ethics vote was needed, according to the German Research Foundation, DZA did not pursue one. All data were collected, anonymised and processed in accordance with strict data protection regulations, to protect participants when working with sensitive mental health information. For further information on DEAS, including methodology, data, findings and related publications, please visit the DZA homepage.^12,14^

### Dependent variable

As the dependent variable, we analysed the depressive symptoms of study participants using a 15-item version of Radloff’s Center for Epidemiologic Studies Depression Scale (CES-D).^15,16^ CES-D is a self-report measure of depression with a range in scores of 0–45, with higher values reflecting more depressive symptoms. The participants were asked 15 questions about their feelings and thoughts over the past week. Each item ranged on a 4-point scale: 1 = rarely or not at all (<1 day); 2 = sometimes (1−2 days); 3 = occasionally or a moderate amount of time (3−4 days); and 4 = most or all the time (5−7 days).^17^

### Independent variable

This study employed a single item to assess subjective under-challenge and overload within the context of participants’ occupations. Participants were asked to evaluate their perceived job adequacy concerning current work demands using the statement: ‘Do you think that you are properly qualified for your job, given the current demands of your work? In view of your qualifications, do you feel…’ Responses were rated on a scale ranging from 1 (under-challenged) to 3 (overloaded).^13^ Given the absence of standardised psychometric instruments for quantification of this construct, we utilised the question above to measure subjective under- and overload at work-given qualification. This methodology resonates with similar formulations in multiple questionnaires designed to assess workplace stressors, e.g. ‘Mental stress – recognising and eliminating risks’.^18^

### Covariates

In alignment with previous research,^3,19–22^ we selected and adjusted for different time-varying covariates. In terms of socioeconomic factors, these were adjusted for age (years), marital status (single, divorced, widowed, married and living separated from spouse, married and living together with spouse), as well as net household income (in euros). Regarding health-related factors, we adjusted for self-rated health, a subjective measure of a person’s overall health status (ranging from 1 = very good to 5 = very bad). Moreover, we adjusted for a count of chronic diseases (ranging from 0 to 11), including cardiac and circulatory disorders; bad circulation; joint, bone, spinal and back issues; respiratory concerns; stomach and intestinal problems; cancer; diabetes; gall bladder, liver and kidney problems; urogenital problems; eye or vision impairment; and ear or hearing problems.

We used the time-constant factor of gender (men/women) to stratify the linear fixed-effects regressions. For descriptive purposes, we utilised education levels categorised according to the International Standard Classification of Education (ISCED-97), distinguishing between low (0–2), medium (3–4) and high (5–6) levels of education.^23^

### Statistical analysis

We performed all statistical analyses using Stata version 17.0 for Windows (StataCorp LLC, College Station, Texas, USA; https://www.stata.com), with statistical significance set at *P* < 0.05. To characterise the data, we conducted descriptive statistical analyses then investigated the correlation of our variables and analysed longitudinal patterns. Therefore, we employed multiple linear fixed-effects regressions to investigate the longitudinal association between the perceived occupational challenge and the occurrence of depressive symptoms. Because both perceived occupational challenges and depressive symptoms were measured in the same survey wave, it cannot be definitively determined whether overload precedes depressive symptoms. In the fixed-effects regressions, we adjusted for the identified time-varying covariates.^22^

Fixed-effects regression is commonly used in panel data analysis to include changes within individuals over time, such as in occupational challenges, from one wave to another. The main advantage of fixed-effects regression is that it can provide consistent estimates even when time-constant factors – observed and unobserved – are associated with predictors.^24^ Consequently, only time-varying factors, such as self-rated health or chronic disease count, can be utilised as explanatory variables in fixed-effects regressions as main effects. Because time-constant factors, such as gender, cannot be used as main effects, we stratified the fixed-effects regression by gender.

## Results

### Sample characteristics

In total, 4362 individuals were included in the study. For those surveyed in 2008 alone (*n* = 2997), their characteristics are outlined in Table 1. The average age was 51.1 years (s.d. 6.2), ranging from 40 to 64 years. Of these individuals, 1540 (51.4%) were male. At baseline, 2203 (73.6%) participants reported being married and living with a spouse. Most participants had an intermediate level of education (50.9%, *n* = 1524), followed by a high level (45.2%, *n* = 1354), while a minority had a low educational level (4.0%, *n* = 119). The monthly mean net household income of study participants (in euros) was 3207.2 (s.d. 2282.1). On average, individuals had 1.6 chronic diseases (s.d. 1.4) and self-rated health was 2.2 (s.d. 0.8). The mean score for depressive symptoms was 5.7 (s.d. 5.4). Further details can be found in Table 1.

**Table 1:** Overload and under-challenge in occupation: baseline characteristics Table 1 long description.

Demographics	Under-challenged, mean (s.d.) or *n* (%)	Challenged appropriately, mean (s.d.) or *n* (%)	Overloaded, mean (s.d.) or *n* (%)	Total, mean (s.d.) or *n* (%)
	*n* = 466	*n* = 2399	*n* = 132	*n* = 2997
Age, years	50.8 (6.6)	51.2 (6.2)	51.8 (5.7)	51.1 (6.2)
Gender				
Male	219 (14.2%)	1259 (81.7%)	62 (4.0%)	1540
Female	247 (17.0%)	1140 (78.2%)	70 (4.8%)	1457
Marital status				
Married and living together	305 (13.8%)	1799 (81.7%)	99 (4.5%)	2203
Married and living separately	13 (21.7%)	44 (73.3%)	3 (5.0%)	60
Divorced	75 (21.4%)	257 (73.4%)	18 (5.1%)	350
Widowed	18 (20.5%)	64 (72.7%)	6 (6.8%)	88
Single	53 (18.2%)	233 (79.8%)	6 (2.1%)	292
Educational level^a^				
Low	13 (10.9%)	100 (84.0%)	6 (5.0%)	119
Intermediate	247 (16.2%)	1212 (79.6%)	65 (4.3%)	1524
High	206 (15.2%)	1087 (80.3%)	61 (4.5%)	1354
Monthly net household income in €	2794.5 (1958.0)	3312.5 (2362.6)	2808.8 (1612.5)	3207.2 (2282.1)
Number of chronic diseases	1.6 (1.4)	1.5 (1.4)	1.9 (1.5)	1.6 (1.4)
Self-rated health^b^	2.3 (0.8)	2.2 (0.8)	2.5 (0.8)	2.2 (0.8)
Depressive symptoms^c^	6.7 (5.9)	5.3 (5.2)	8.4 (6.4)	5.7 (5.4)

a. The International Standard Classification of Education (ISCED-97) provides a framework for the categorisation of educational status, achievements and qualifications into internationally recognised levels, ranging from 0 to 6. A person with ISCED 0–2 is considered to have a low level of education, while ISCED 3–4 is intermediate and ISCED 5–6 is high.^23^

b. Self-rated health is a subjective measure of a person’s overall health status, ranging from very good (1) to very bad (5).^13^

c. The Center for Epidemiological Studies Depression Scale (CES-D) is a self-report measure of depression with a scoring range of 0–45, with higher values reflecting more depressive symptoms.^17^

Regarding feelings of being under-challenged, 247 women (53.0% of all under-challenged participants) reported such sentiments, compared with 219 men (47.0%). Conversely, 70 women (53.0%) and 62 men (47.0%) reported feeling overloaded. Most participants – 1140 women (47.5%) and 1,259 men (52.5%) – perceived the challenge level in their occupation as exactly right.

### Regression analysis

#### Total sample

The results of multiple linear fixed-effects regressions are shown in Table 2 (total sample; stratified by gender; and with interaction terms). Intra-individual transitions, from ‘challenged appropriately’ to ‘under-challenged’ for occupation were not significantly associated with changes in depressive symptoms. In contrast, intra-individual transitions from ‘challenged appropriately’ to ‘overloaded’ were significantly associated with increases in depressive symptoms (*β* = 1.39, 95% CI: 0.38 to 2.40, *P* < 0.01).

**Table 2:** Linear fixed-effects regression, with occupational challenge as the dependent variable and depressive symptoms over time, adjusting for potential time-varying covariates Table 2 long description.

Depressive symptoms				
Variables	Total sample, *β* (95% CI)	Men, *β* (95% CI)	Women, *β* (95% CI)	Total sample, with interaction, *β* (95% CI)
Under-challenge (reference: challenged appropriately)	0.13 (−0.46 to 0.72)	0.39 (−0.37 to 1.14)	0.04 (−0.90 to 0.82)	0.33 (−0.41 to 1.08)
	*P* = 0.66	*P* = 0.32	*P* = 0.92	*P* = 0.38
Under-challenge × gender^a^				−0.36 (−1.51 to 0.78)
				*P* = 0.54
Overload (reference: challenged appropriately)	1.39 (0.38 to 2.40)	2.16 (0.70 to 3.60)	0.79 (−0.58 to 2.16)	2.13 (0.67 to 3.59)
	***P*** ** < 0.01**	***P*** ** = 0.004**	*P* = 0.26	***P*** ** = 0.004**
Overload × gender^b^				−1.30 (−3.31 to 0.71)
				*P* = 0.20
Time-varying covariates	✓	✓	✓	✓
Number of observations	7487	3668	3819	7487
Number of individuals	4362	2169	2193	4362
*R* ^2^	0.163	0.152	0.164	0.16

Results of linear fixed-effects regressions with unstandardised *β* coefficients. Time-varying covariates include age, marital status, net household income, self-rated health and a count of chronic diseases; ‘×’ denotes interactions.

a. Interaction term 1: women (reference: men) × under-challenged (reference: challenged appropriately).

b. Interaction term 2: women (reference: men) × overloaded (reference: challenged appropriately).

*P*-values in bold indicate statistical significance **P < 0.01.

#### Stratified by gender

When stratified by gender, intra-individual transitions from ‘challenged appropriately’ to ‘under-challenged’ for occupation were not significantly associated with changes in depressive symptoms among male individuals (*β* = 0.39, 95% CI: −0.37 to 1.14, *P* = 0.32). However, intra-individual transitions from ‘challenged appropriately’ to ‘overloaded’ among men were significantly associated with changes in depressive symptoms (*β* = 2.16, 95% CI: 0.70 to 3.60, *P* = 0.004).

Among women, the average within-person change from ‘challenged appropriately’ to ‘under-challenged’ was not significantly associated with changes in depressive symptoms (*β* = 0.04, 95% CI: −0.90 to 0.82, *P* = 0.92). Intra-individual transitions from ‘challenged appropriately’ to ‘overloaded’ among female individuals were also not significantly associated with changes in depressive symptoms (*β* = 0.79, 95% CI: −0.58 to 2.16, *P* = 0.26). The corresponding interaction terms (occupational challenge **×** gender) did not achieve statistical significance. Please see Table 2 for further details.

## Discussion

This longitudinal study aimed to investigate the association between subjective work challenges and depressive symptoms – also stratified by gender – using a fixed-effects regression model. Our research extends the very few existing cross-sectional studies by utilising longitudinal data from a large, nationally representative sample. Our key findings revealed that intra-individual transitions from being appropriately challenged to overloaded in the ‘occupation, given job qualification’ were significantly associated with increased depressive symptoms among the total sample. Even moderate increases in depressive symptoms can affect daily functioning and work performance, highlighting the importance of addressing overload in the occupation. Our study found similar associations between overload and depressive symptoms among men, but not among women, whereas intra-individual transitions from being appropriately challenged to under-challenged were significantly associated neither with changes in depressive symptoms in the total sample, nor when stratified by gender. Although significant research has been conducted on the relationship between job dissatisfaction or job strain and the risk of psychological diseases, it remains unclear how completion of tasks for which workers do not feel qualified impacts the development of these diseases. Several studies have found that low job satisfaction is significantly linked to depressive symptoms and anxiety.^6^ Job satisfaction is measured mainly by either a single-item question or assessing an employee’s satisfaction with various job components, such as whether they find their job interesting, have good relationships with managers and colleagues, receive a high income, have the freedom to work independently and have clear career advancement opportunities.^6,25–27^ Individuals experiencing high job strain – which is characterised by high demands and low work control – are also more likely to develop clinical depression.^7^ The Swedish Demand-Control-Support Questionnaire (DCSQ) is a validated tool often used to study job strain.^28^ However, this questionnaire does not include questions regarding psychological demands based on qualifications. Although overload can be considered a factor for increased job strain, the DCSQ does not cover this. Nonetheless, our findings linking overload to depressive symptoms suggest that it could be considered a component of increased job strain, supporting the evident association between job strain and depression. Even if challenges stemming from professional qualifications are regarded as a component of psychological demands, examining these as an independent variable remains valuable. Despite some studies incorporating high demand or work overload into their composite outcomes, none have explored challenges because of over- or under-qualification as distinct factors.^6,7,11^ These, however, might particularly affect the negative work-related stress and, thus, the occurrence of depression. For instance, while high demands may not necessarily be stressful for a well-qualified and satisfied worker, they could become distressing if coupled with feelings of under-qualification. We recommend future research to examine this in more detail.

Our study results are particularly interesting in light of the gender-specific differences found; the non-significant interaction may be explained largely by a lack of statistical power. The literature suggests that women have a more inadequate stress adaptation and a higher prevalence of depressive disorders.^3,29^ One possible explanation is that when work is considered more critical for one’s satisfaction and fulfilment in life than in other areas, the absence of such satisfaction can significantly affect one’s mental health.^30^ Recent data indicate that female employment in Germany has increased in the past few years, resulting in a decrease in gender-based disparities in employment ratios. Nevertheless, women tend to be employed in part-time jobs more frequently than men, which means that men still work more hours overall.^31^ As a result, men may view work as a more central aspect of their lives than women.^32^ Therefore, men may also be at a greater risk of experiencing depressive symptoms due to work-related challenges compared with women.

In a Canadian study, researchers discovered that men who deal with high demands and low control at work are more prone to experiencing major depression and anxiety disorders.^33^ Conversely, for women, this specific combination tends to be linked more broadly to various forms of depressive or anxiety disorders. Furthermore, that study highlighted that job insecurity is positively linked with major depression in men, while no such correlation was found among women.^33^ Therefore, one could argue that, if men feel overloaded at work, they may also be at a greater risk for depression due to fear of job loss. Research also indicates that depression among male workers is greater than the national average, especially in male-dominated industries, where rates of depression can vary.^34^ In this context, breakdown of the areas in which our study participants were employed is also exciting and could be addressed in future studies. In addition, the differences in how men and women cope with work-related stress may also help to explain our research findings. Women tend to use adaptive coping strategies, such as seeking support from family and friends, to deal with stress. On the other hand, men are more likely to use maladaptive and avoidance strategies when faced with stressful situations.^35^ These findings suggest that women adopt healthier and potentially more effective coping mechanisms when dealing with overload at work, compared with men. All the foregoing suggests that unexplored factors such as individual coping strategies, social support networks and variations in stress perception may underlie gender differences.^36^ Therefore, further research must thoroughly examine these variables in future studies.

Our study contradicts one previous study which showed that subjective under-challenge at work is positively associated with depressive symptoms, mediated by life satisfaction.^8^ This relationship between under-challenge and depressive symptoms is consistent across genders in the above study. Other studies indicate a connection only between under-challenge and mood, not depression.^37,38^ However, conducting further research on this topic is essential to understand better why our longitudinal study produced outcomes different from those of previous cross-sectional studies. One plausible explanation could be that overload may result in longer working hours, whereas individuals experiencing under-challenge may complete their tasks within regular hours, thus affecting differently the perception of stress and its impact on depressive symptoms. Of note, long working hours are positively associated with depressive symptoms.^39,11^

### Strengths and limitations

We should consider some of this study’s strengths and limitations when interpreting our current findings. First, this is one of few studies on overload and under-challenge given job qualifications, and their impact on mental health. Second, we used a large, nationally representative sample. Moreover, we utilised longitudinal data and fixed-effects regressions, which significantly reduced the problem of unobserved heterogeneity, a challenge that often arises when using observational data. However, the possibility of reverse causality (i.e. changes in depressive symptoms may also contribute to changes in the perception of occupational challenges) cannot be dismissed. Future research in this area is thus required. We used a single question item to determine whether the participant’s occupation was subjectively overloading or under-challenging, because no validated tools are currently available to measure this variable directly. Although single-item measures may not capture constructs as comprehensively as multi-item scales, research indicates that thoughtfully phrased single items can still provide psychometrically valid evaluations of work stressors.^40^ However, all other variables were collected using standardised and validated methods. It is worth noting that the DEAS study has revealed a slight sample selection bias: for instance, participation rates may be lower among residents of large cities. Nonetheless, such effects are minor, and the distribution of sociodemographic characteristics is reasonably similar to that in Germany.^12^

### Implications

Based on our study, middle-aged men facing overload at work exhibit higher depressive symptom scores. Efforts to prevent overload may, therefore, be beneficial in avoiding mental health diseases. Although our study did not find heightened depressive symptoms among individuals experiencing under-challenge, there may be implications for efficiency and task allocation. Further research from diverse populations is needed to confirm these findings. Investigation of whether the COVID-19 pandemic had affected work-related challenges would be interesting. Given the significance of family planning in this age group, analysis of work challenges and their association with depressive symptoms among individuals aged 18–39 years, particularly gender-specific differences, is also essential. By addressing work-related stress and reducing overload, we can potentially prevent depression onset, emphasising the importance of supporting individuals at risk.

## Data Availability

The data used in this study are third-party data. The anonymised data-sets of the German Ageing Survey (DEAS) (1996, 2002, 2008, 2011, 2014, 2017, 2020, 2020/2021, 2023) are available for secondary analysis. The data have been made available to scientists at universities and research institutes exclusively for scientific purposes. The use of data is subject to written data protection agreements. Microdata from DEAS are available free of charge to scientific researchers for non-profitable purposes. The Research Data Centre at the German Centre of Gerontology (DZA) provides access and support to scholars interested in using DEAS for their research. However, for reasons of data protection, signing a data distribution contract is required before data can be obtained. For further information on the data distribution contract, please see https://www.dza.de/en/research/fdz/access-to-data/application (accessed 24 September 2025).

## Table 1 Long description

The table presents baseline characteristics of 2997 individuals surveyed in 2008. It has 11 rows and 5 columns. The columns are labeled Demographics, Under-challenged, mean (s.d.) or n (%), Challenged appropriately, mean (s.d.) or n (%), Overloaded, mean (s.d.) or n (%), and Total, mean (s.d.) or n (%). The rows include Age, Gender, Marital status, Educational level, Monthly net household income, Number of chronic diseases, Self-rated health, and Depressive symptoms. Each row provides specific data points for each category. For example, the average age is 51.1 years with a standard deviation of 6.2. The table also includes percentages and means with standard deviations for various demographic and health-related characteristics.

Navigate back to Table 1.

## Table 2 Long description

A table with depressive symptoms as the focus, showing the results of linear fixed-effects regression. The table has 4 rows and 5 columns. Column headers are Variables, Total sample, Men, Women, and Total sample with interaction. Row labels include Under-challenge, Under-challenge x gender, Overload, and Overload x gender. The table presents beta values with 95% confidence intervals and P-values for each variable across different groups. Notable trends include a significant association between overload and increases in depressive symptoms in the total sample and among men.

Navigate back to Table 2.
